# Moria or Mania? Manic symptoms as the clinical manifestation of glioblastoma recurrence: a case report

**DOI:** 10.1192/j.eurpsy.2023.1103

**Published:** 2023-07-19

**Authors:** F. Mayor Sanabria, M. E. Expósito Durán, M. Fernández Fariña, C. E. Regueiro Martín-Albo, M. Paz Otero, I. Alberdi Páramo, B. Rodado León

**Affiliations:** Instituto de Psiquiatría y Salud Mental, Hospital Clínico San Carlos, Madrid, Spain

## Abstract

**Introduction:**

Up to 50% of patients with brain tumors experience psychiatric symptoms, and rates up to 80% have been reported in malignant neoplasms such as glioblastoma multiforme (GBM). Still, clinical presentation as mania-like syndromes is a rare phenomenon, mainly occurring when frontal structures are compromised.

We present the case of a 42-year-old woman who was admitted to our hospital due to manic symptoms coinciding with a recurrence of a bifrontal GBM, for which she underwent surgery 5 months prior.

**Objectives:**

1) To describe the clinical particularities of this case, focusing on the differential diagnosis.

2) To review the association between manic symptoms and frontal dysfunction caused by brain tumors, with special interest on GBM.

**Methods:**

A review of the patient’s clinical history and complementary tests performed was carried out. Likewise, we reviewed the available literature in relation to manic symptoms related to brain tumors.

**Results:**

The patient’s GBM recurrence presented with late onset symptoms of mania, including euphoric mood, increased spending, ideas of grandiosity and hyper-religiosity. She had no previous psychiatric history but, interestingly, she had an extensive affective burden in her family, with 4 consummated suicides. However, she also presented other clinical signs, such as disorientation, perseveration, mild memory impairment and stereotyped motor behaviors, that pointed to relevant frontal lobe dysfunction, suggesting Moria as a possible contribution for the symptoms described.

Manic symptoms in the context of brain tumors appear in 7-15% of patients with psychiatric symptoms, usually associated with right frontal dysfunction (75% of cases). Bifrontal affectation, such as this patient, is only described in 6% of cases. Although fast growing, malignant tumors have been associated with higher rates of psychiatric symptoms, no correlation has been described between these and brain tumor histology.

**Conclusions:**

- The presence of atypical manic symptoms, such as those presented in this case, should raise clinical concern for secondary mania.

- Moria shares similarities with mania, including mood elevation, tendency to hilarity or hyper-sexuality, that may hinder diagnosis of patients with frontal dysfunction.

- This case outlines the difficulties in making a differential diagnosis in patient with both manic and neurological signs, and highlights the implication of frontal structures in the development of manic symptoms.

**Disclosure of Interest:**

None Declared

